# Hantavirus Public Health Outreach Effectiveness in Three Populations: An Overview of Northwestern New Mexico, Los Santos Panama, and Region IX Chile

**DOI:** 10.3390/v6030986

**Published:** 2014-02-27

**Authors:** Marjorie S. McConnell

**Affiliations:** Department of Biology, University of New Mexico, MSC 03 2020, 1 University of New Mexico, Albuquerque, NM 87131, USA; E-Mail: mmcconnell@lternet.edu; Tel.: +1-505-563-0320; Fax: +1-505-277-2541

**Keywords:** hantavirus, public health, infectious disease knowledge, behavior change, social epidemiology

## Abstract

This research compared the effectiveness of Hantavirus Pulmonary Syndrome (HPS) outreach programs in New Mexico, Panama, and Chile. Understanding the role of human demographics, disease ecology, and human behavior in the disease process is critical to the examination of community responses in terms of behavior changes. Attitudes, knowledge, and behavior across three populations were measured through the implementation of a self-administered questionnaire (N = 601). Surveys implemented in Chile and Panama in 2004, followed by northwestern New Mexico in 2008, attempted to assess knowledge and behavior change with respect to hantavirus in high- and lower-risk prevalence areas during endemic periods. While levels of concern over contracting hantavirus were lowest in New Mexico, they were highest in Panama. Respondents in Chile showed mid-level concern and exhibited a tendency to practice proper cleaning methods more than in New Mexico and Panama. This indicates that public health messages appear to be more effective in Chile. However, since negative behavior changes, such as sweeping and vacuuming, occur at some level in all three populations, improved messages should help decrease risk of exposure to HPS.

## 1. Introduction

In May 1993, an outbreak of a seemingly new, deadly disease hit the Four Corners region of Northwestern New Mexico. Just 19 days after the first case of Hantavirus Pulmonary Syndrome (HPS) was observed in New Mexico, Centers for Disease Control (CDC) and an interdisciplinary team of University of New Mexico scientists identified the cause of the disease outbreak as a previously unknown hantavirus which eventually became known as Sin Nombre Virus (SNV) [1]. New World hantaviruses such as SNV prove to be highly pathogenic, causing in humans a potentially fatal respiratory condition known as Hantavirus Pulmonary Syndrome (HPS) [1,2]. In 1995, two years following the North American outbreak of HPS, another hantavirus emerged in the Aysen (Region XI) of southern Chile [3]. Similar to Sin Nombre Virus, this hantavirus was rodent-borne, caused HPS, and was subsequently named Andes Virus (AND) [3]. In December 1999, Panama’s first case presented in the community of Tonosi, Los Santos Region and subsequently named Choclo Virus (CHOC) [4]. It is widely recognized that human populations become infected indirectly through exposure via inhalation of virus particles in dust contaminated with rodent droppings and urine [5]. There is no known medical intervention for HPS, nor is there a vaccine.

Because hantavirus transmission is believed to be rodent-borne, public health messages are designed to lower exposure risk by reducing potential contact with rodents [5,6]. Positive prevention measures include rodent-proofing activities such as repairing screens and holes in walls, trapping, wearing rubber gloves to clean, and by disinfecting and mopping with common household detergents such as bleach or disinfectant spray [6,7]. In peridomestic areas, moving woodpiles away from the house, removing trash, and keeping lids on garbage cans are recommended methods of keeping rodents away from the home [5,6]. These recommended methods turn out to be inexpensive and effective. This may benefit not only those in high-risk rural areas where hantaviruses pose a particular endemic problem, but wherever hantaviruses appear as a threat to human health to populations with limited resources. 

This work expands the understanding of how social context influences hantavirus transmission by evaluating the role of human behaviors in the Sin Nombre Virus/HPS, Choclo Virus/HPS and Andes Virus/HPS, and processes in the United States, Panama, and Chile, respectively. This study follows the traditions of social epidemiology, a major concentration and substantial growth area of public health studies of many behavior-related diseases. To date, no other formal evaluations on the effectiveness of hantavirus outreach had been conducted across populations, although the need for such investigation is called for by public health officials in the United States [8] and the Pan American Health Organization [9]. Given the worldwide threat of hantaviruses and an estimated 200,000 new cases of both Hemorrhagic Fever with Renal Syndrome (HFRS) and HPS appearing each year [2], evaluating outreach efforts will help enable public health officials to design efficient and effective programs in the future. A careful analysis of the behavioral aspects and reported change of the disease process could be vitally important if not indispensable to designing effective prevention programs as well as improving programs already in place.

Specific aims of this project were: (1) to assess whether the exposure to public health information makes a difference in terms of knowledge gained and responses to the information; (2) to assess concern about hantavirus or self and family within three different human populations of New Mexico, Panama, and Chile; and (3) to assess whether concern translated to risk reduction behavior in terms of appropriate preventive practices such as mopping and disinfecting, rather than inappropriate practices such as sweeping or wearing a dusk mask while performing routine household cleaning activities [10]. 

Outreach programs vary in New Mexico, Panama, and Chile. Programs in New Mexico tend to be more *ad-hoc* and reactive to potential increases in human incidence by environmental modeling as predictors. Panama also relies on more passive and *ad-hoc* programs, but compared to New Mexico, Panama utilizes a slightly higher outreach platform to rural communities with information dissemination through informational posters in local communities and health clinics. Chile, however, invested larger resources to implement an extensive two-step on-going program based upon outbreak and endemic periods. [10] 

During the initial Four Corners hantavirus outbreak in 1993–1994, the New Mexico State Department of Health and the Centers for Disease Control’s (CDC) first step for disseminating information was to set up a “hantavirus hotline”. During peak crisis time, the hotline fielded more than 1,000 calls per hour [11,12]. Second, educational materials were developed for medical personnel to recognize and successfully triage potential hantavirus cases, and for transporting patients to the University of New Mexico medical center critical care unit. Educational materials were also developed by the CDC to be made available upon request to the public. For example, in 1993 and 2002, the CDC published recommendations on hantavirus risk reduction via specific instructions on proper household cleaning, rodent reduction, cleaning up infested areas, and precautions to take with regard to occupational hazards plus recreational activities such as camping or hiking [6]. Lastly, the CDC created a prevention video that was disseminated upon request. Today, prevention measures are disseminated through individual state health department websites that also have links to the CDC for further information. Official outreach programs remain passive and *ad-hoc*, via websites that can be referenced by the public for case information, proper cleaning methods, and hantavirus etiology. Finally, as cases occur, local newspapers and regional television reporting identifies incidence, and the status of the latest victim, but not beyond a sound-bite time frame. [10] 

Although the first case of hantavirus in Chile appeared in 1995 in Cochamo, sporadic cases occurred until the outbreak of 1997–1998 occurring in Aysen Region XI [3] During the official outbreak, health ministry officials implemented specific communication strategies to help calm a concerned population and provide prevention information [9]. These actions, in part, were based upon prior knowledge of hantaviruses identified in New Mexico combined with the sporadic cases from 1995–1997. Thereafter, a number of actions were implemented at the community level by the health ministry with media support: (1) daily hantavirus reports were issued; (2) information was disseminated through the schools, buses, and other public places and (3) a scientific conference was held for journalists, wherein the media produced a “hantavirus radio day” which linked the health specialists to the population [9]. Secondly, and more importantly, a two-stage “Hantavirus Prevention National Campaign” was designed and implemented with general and summer objectives [9]. General prevention strategies promoted proper in-home cleaning habits while broadcast media support included rural broadcasting radio and television. Healthcare workers and hospital professionals then distributed written media posters and informational notebooks to rural and semi-urban zones and schools [9]. Summer HPS prevention activities disseminated information on how to remain safe during typical summer activities such as camping and how to properly clean summer cabins. Similar to the general prevention strategies, broadcast media support included radio and television messages that promoted proper cleaning methods for cabins and safe camping practices, posters, and prevention information for national park employees [9]. Finally, the Chilean Ministry of Health’s outreach plans for non-outbreak periods included keeping the population informed about new cases via an internet webpage, and cautioning the public to maintain proper domestic hygiene habits. The Health Ministry also continued to air television commercials geared toward making rural residents aware of risks of hantavirus and the benefits of easy, low-cost prevention measures [9]. 

Because the concept of consistent messages is critical to the Chilean two-step campaign, and to keep the public aware of the dangers of hantavirus exposure, non-outbreak periods also carry messages to the public. During these periods, the Chilean Ministry of Health informs the public of new cases via an internet webpage and cautioning the public to maintain proper domestic hygiene habits. To further the public education of hantavirus awareness, prevention activity days are staged in regions such as Bio-Bio and Aysen that have higher endemic incidence. Co-sponsored by the Ministry of Health, the Forestry Department and industry, prevention activity days are created as family-friendly events which feature people costumed as predatory animals that are good for the environment such as owls that also look like forest rangers. During these promotional activities, calendars that feature proper cleaning methods are distributed, along with posters that also describe the benefits of wild animals such as owls and snakes that prey on rodents. 

Finally in Panama, during the initial 1999–2000 outbreak, Panamanian Ministry of Health officials established suspected hantavirus case surveillance and a telephone hotline was established to inform the public of proper prevention measures. Educational materials were distributed to general public, and medical providers were educated to recognize potential cases and to also educate the public. Further outreach included posters in health clinics, and continued medical personnel training. As in the United States passive, *ad-hoc* outreach through a webpage informs the public via reports and overall plans to protect the general health of the population. As cases occur, public health officials continue to brief local health clinics and rodent trapping crews are dispatched to outbreak areas to determine sero-prevalence and the potential of another outbreak [13,14]. Finally, the Panamanian Ministry of Health, in cooperation with the Hantavirus Research Project through the National Institutes of Health [15] has continued to engage in sero-prevalence research activities to determine not only rodent population densities but also with the public to determine who has been exposed to hantavirus. This current research, however, does not address specific outreach programs, nor does it measure the effectiveness or public behavior changes. [15] 

The above descriptions elucidate the varied programs each country implemented during initial outbreak periods and later efforts to keep the public informed. In all three countries, the common denominator was the rapid responses to the critical periods: that of hotlines to answer questions, public health officials working with the media to disseminate information, and medical personnel training to triage potential cases and identify patients that require immediate attention. Beyond the critical periods, however, the United States and Panama engage in passive *ad-hoc* outreach prevention programs through webpages the general public can access. However, 2004 through 2009, Chile invested approximately 600 million pesos (approximately USD 1.5 M) specifically toward hantavirus prevention programs [10]. These prevention and training programs as described above, actively engage in extensive outreach by committing to seasonal messages distributed accordingly, and to engage industry and other governmental organizations in creating awareness activities, including promotional information. It is critical to identify if the more extensive outreach in Chile is effective in engaging its citizens in proper cleaning methods, in comparison to citizens in Panama and Chile. 

To underscore the importance of the problem in at-risk populations, the incidence of reported cases is shown in Table 1. While Panama and Chile are experiencing higher case and mortality rates per 100,000 than the United States, approximately one third of the patients who contract HPS in the United States and Chile do not survive. There may be several reasons for these differences, such as rodent population densities, human interactions with the environment, and virus virulence between SNV, CHOC, and AND, but the issue and subject of this project is how outreach messages intended to inform the public are being received, and if the messages are effective. 

**Table 1:** Incidence of identified cases in the United States, Panama, and Chile.

Cases	United States of America	Panama	Chile
	585 (1993–2012) [16]	176 (2000–2012) [17]	768 (2001–2012) [18]
Case Rate per 100,000	0.186	5.014	4.60
Mortality Rate per 100,000	0.066	0.911	0.162
Death/Case Rate per 100	35.50	18.20	35.24
Age (years)	6–83 (mean 37)	1–84 (mean 43)	<1–80 (mean 33)

## 2. Methods

### 2.1. Study Sites

This study measured knowledge and behavior responses by comparing attitudes and behavior toward HPS in three countries, with nine survey sites (three in each country) during an endemic period. After consulting with public health officials in New Mexico, Panama, and Chile, each in-country site was chosen based upon HPS reported incidence and geographic locations relative to high- and lower-risk sites. Each country comprised three sub-sites that best represented two high-risk areas of contracting HPS and one lower relative risk area that acted as a control site (Table 2). These sites were determined by reviewing similar outbreak rates and geographical locations in relation to lower-risk areas. 

**Table 2:** Country site selection matrix according to high-risk and lower-risk incidence.

Country site	High-risk	Lower-risk
New Mexico (n = 200)	Gallup, Farmington (n = 133)	Grants (n = 67)
Panama (n = 200)	Tonosi, Pocri (n = 133)	Jaguito/El Roble (n = 67)
Chile (n = 201)	Melipeuco, Curacautin (n = 134)	Temuco (n = 67)

### 2.2. Procedures

In total, 601 surveys were collected from northwestern New Mexico (2008), Los Santos Panama (2004), and Region IX Chile (2004). Knowledge and behavior, both positive and negative, were measured through the implementation of a self-administered 28 question survey in local health clinics and community neighborhoods. The intent was to: (1) determine general hantavirus knowledge in high- and low-prevalence areas; and (2) to determine what changes in preventive practices have occurred in those areas. In terms of knowledge, it was assumed there was no difference in terms of hantavirus knowledge between high and low prevalence areas. There was also the assumption that with respect to positive behavior changes to reduce risk of exposure to hantavirus, there was no difference between the more urban control sites and the rural poor, who represent the high-risk groups. Although the hypotheses were designed as null, it was reasonable to expect some differences between knowledge and positive/negative behavior changes. Additionally, overall outcomes were expected to be better in Chile in terms of knowledge and positive behavior changes because more extensive outreach as described above is conducted in Chile than in Panama or New Mexico [9].

Institutional Review Board (IRB) approvals were obtained in each country prior to the surveys being implemented. In the United States, the University of New Mexico IRB approved the project continuously from the implementation of the pilot project in 2000 through the conclusion of the dissertation project in 2009. In Panama, IRB approval was obtained through the Gorgas Institute in conjunction with the Panamanian Minister of Health. In Chile, the Ministry of Health, through the Center for Infectious Diseases, approved the implementation of the surveys. To assure each respondent the necessary human subject protections, each survey began with the voluntary disclaimer, “Your participation is voluntary and anonymous. You can choose not to participate or not complete the survey and the decision will not affect your medical attention.” Incentives were not offered to the respondents in any country or site for their participation in the study. The pilot study conducted during Summer 2000 in northwestern New Mexico determined that time to complete the self-administered survey was approximately 5–10 min, thus utilizing an efficient way to capture information. 

### 2.3. Project Measures and Survey Instrument

Table 3 below outlines the major variables in the survey: antecedent survey sites, independent socio-economic variables, mediating environmental and predicating variables, and finally dependent variables of knowledge and behavior responses. 

### 2.4. Survey Instrument Details

As described above, antecedent survey sites in each country survey were selected after consulting with public health officials in New Mexico, Panama, and Chile, and based upon HPS reported incidence and geographic locations relative to higher- and lower-risk areas. The initial portion of the survey instrument measured if, when, and how the respondent first heard about hantavirus, whether through the media, family, television, or some other method. Responses to public service announcements, if heard, were measured through what would be an appropriate response such as “check for mice” or a misinformed response such as “get a flu shot” or “took vitamins” or no response at all. If the respondent had not heard of hantavirus, he/she was instructed to skip questions to the socio-economic variables at the end of the survey. 

Mediating environmental variables included: What is the respondent’s length of time in the area, what type of home does he/she live in, age of the home, own/rent home and what type of biome do they live? In particular, the biome was requested, because ecological studies have revealed that sero-prevalence of the agent in the reservoir is higher in certain biomes than others where the risk is lower [19]. Observation of rodents in the home or garage was also asked, as a measure of cue to action for behavior change. 

**Table 3:** Antecedent, independent, mediating, and dependent variables [10].

Antecedent	Independent Variables	Mediating Variables	Dependent Variables	
Survey Sites	Socio Economic Variables	Environmental and predicating	Knowledge responses	Behavior responses
Chile	Q. 21 Residence	Q. 11 Length of time in area	Q. 1 If heard of hantavirus	Q. 17 Levels of change
Temuco (control)				How cleaning habits changed:
Melipeuco	Q. 22 Sex	Q. 12 Type of home	Q. 2 When heard of hantavirus	Q. 18 Mop more
Curacautin				Q. 18 Sweep/vacuum more
	Q. 23 Age Group	Q. 13 Age of home	Q. 3 How heard of hantavirus	Q. 18 Disinfect more
Panama				Q. 18 Set traps
Jaguito (control)	Q. 24 # of minor children	Q. 14 Own/rent home		Q. 18 Wear rubber gloves
Pocri			Q. 6 Know someone who has contracted hantavirus?	Q. 18 Wear dust mask
Tonosi	Q. 25 Ethnicity	Q 15 Biome		Q. 18 Wear ventilator mask
				Purchase more:
NW New Mexico	Q. 26 Household income	Q. 16 Observed rodents in home	Q. 8 How do people catch hps?	Q. 19 Traps
Grants (control)				Q. 19 Poison
Gallup	Q. 27 Occupation		Q. 9 Animals that carry hps	Q. 19 Bleach
Farmington				Q. 19 Dust masks
	Q. 28 Years of Education		Q. 10 Symptoms of hps	Q. 19 Disinfectants
				Q. 19 Ventilator masks
				Improvements:
				Q. 20 Mouse proof home
				Q. 20 Remove trash
				Q. 20 Clean up wood piles
				Q. 20 Cut grass/weeds
				Reactions to HPS
				Q. 4 Response upon hearing about hps
				Q.5 Response to PSA
				Q.7 Concern about catching hps

Dependent variables were divided into two categories of knowledge and behavior responses. Knowledge responses included correct and incorrect choices in the following categories: If, when, and how the respondent learned of HPS, how is HPS contracted, do they know someone who has contracted HPS, what carries hantavirus (including incorrect answers such as insects), and symptoms of HPS. It should be noted that local vocabularies for the rodent reservoir were suggested by in-country review boards. Local vocabularies were thus able to capture the intent of the question to ensure reliability of measurement across the three sites. For example, in Chile and Panama, use of the word “ratones” more accurately described the hantavirus reservoirs for AND and CHOC, whereas in New Mexico, use of the word “mice” was more accurate for SNV. Because the reservoir is rodent-borne, with different rodent species acting as carriers of the specific hantaviruses, using language specific to communities surveyed was deemed appropriate. 

Behavior changes, both positive and negative were then measured. Following the mediating question regarding any direct observations of rodents in the home or surrounding buildings, was a question if behavior had changed and, if so, how much. If so, did the described and measured knowledge of proper cleaning and prevention methods actually show the necessary positive behavior changes. As in other questions, appropriate and inappropriate answers were imbedded into the sections to measure the respondents’ behavior based upon knowledge or mediating circumstance. For example, respondents were asked if they mop (appropriate), sweep/vacuum (inappropriate), and if they wear a ventilator mask (appropriate) or a dust mask (inappropriate). Respondents were also asked if they purchased cleaning supplies, and if so what kind such as bleach or household disinfectant. Final questions regarded the peridomestic area, and if respondents are cleaning the yard in an appropriate manner: (1) mouse-proof; (2) removing trash; (3) cleaning up woodpiles; (4) cutting grass and weeds. If habits had not changed at all, regardless of direct observation of rodents, or media messages, or knowledge level, the respondent was then asked to proceed directly to the socio-economic independent variables. In this way analysis could determine if there were any significant differences between those whose habits have changed or not, by gender, age, ethnicity, income, occupation, and years of education. 

The concept of a single independent variable to identify socio-economic status is so status can be objectively defined, yet each variable also measures a portion of social complexity in terms of which populations may be more susceptible to disease exposure. That stated, the following independent socio-economic variables included: community of residence, gender, age range, number of children under 18 living at home, ethnicity, income range, occupation/activity, and years of formal education achieved. Community of residence identifies the proximity of the respondent’s home to high/low-risk biomes. Age, however, should bear out as the most important variable because average age may be linked to the development of disease [20]. Average patient age in each country, as described in Table 1, suggests the question, what would the population be doing at the median age range that increases risk of exposure? Are the messages reaching the median age range of concern, and if so can they be compared to knowledge and behavior changes in relation to those who actually contract HPS in a particular region [10]. Gender is second in importance because of morbidity/mortality differences. Gender can also be compared for significant differences between male/female in concern for familial health and associated behavior changes. 

Number of children under age 18 provides an indicator of not only household size and relative resources, but index cases are frequently familial. This becomes more important in South America because children are more likely to contract HPS than in North America. Ethnicity may be linked to particular locations that are inhabited by groups of people, engaged in similar activities. How well messages may be received can also be influenced by ethnicity, as different cultural parameters may guide community pressure to perform and behave in prescribed manners. Income also influences living conditions, areas of residence, and resources available for proper household cleanup. While the methods are inexpensive and easy to implement, those with lower income may be more interested in placing food on the table than mopping the floor, for example. Therefore environmental influences may also drive levels of behavior change.

Occupational hazards associated with rural activities such as forestry and agriculture can help identify specific risk groups. Community occupations also identify general community conditions and how particular groups work and live within specific geographic associations with high-risk. Also, those employed in agricultural occupations tend to earn lower wages, experience overall lower nutritional values, or live in substandard, transient housing. All of these factors can lead to a higher susceptibility to HPS exposure, either through actual working activity, or subsequent conditions. Because HPS in the Americas is considered a rural disease and associated with rural activities, each site survey listed occupations appropriate for the area. For example, New Mexico occupation categories included professional, farmer, rancher, and so on, while Chilean surveys deleted rancher and added fisherman or forestry, since those occupations are common. 

Finally, income and years of education ranges were site-specific on the instrument. Because monetary value was confined to each country, income ranges were analyzed in the context of each country. United States Dollars should not be compared to Panamanian Balboas or Chilean Pesos, but does give an indication of poverty/wealth within each survey site. Levels of education are also site-specific because education is different in each country. For example, in the Chilean and Panamanian surveys, it was recommended by the in-country IRB approval committees to add technical schools because post-high school equivalent does not necessarily mean university education. 

### 2.5. Survey Implementation and Analyses

Actual survey implementation, with one exception, occurred in local health clinics as a central point of data collection. In this way, public health officials and medical personnel had direct supervision of data collection efforts through central health ministry approvals as well as a vested interest in the outcomes. However, the nature of hantaviruses in the Americas is that HPS is a rural disease, and therefore, potentially susceptible communities are also rural. Therefore, where data collection at the local clinic was not feasible because the community was very small and traffic through the clinic was sparse, surveys were completed in a nearby neighborhood. In this circumstance, every other household was approached. If no one was home or they were unwilling to participate, the next available house was selected. While this introduces a small bias because of an inconsistency in data collection method (clinic *vs*. neighborhood), it is believed that the differences were minimal, because these very communities were rural and small. Overall, the local communities were very responsive to taking the survey. There were no incentives for participating in the questionnaire, and each respondent was given the opportunity to stop the survey with no repercussions. 

The Chilean data (n = 201) collection occurred during January 2004 in Region IX communities of Temuco, Melipeuco, and Curacautin. The researcher, along with Chilean health officials and medical personnel, proceeded into the clinics after local approvals and distributed the instrument to waiting room people. Each surveyor wore appropriate identification cards and uniforms to establish trust. During the Chilean collection efforts, Melipeuco was the only community where the local clinic was not feasible for data collection due to the small community size and low clinic volume. Only one household respondent refused to take the survey, so the next available house was selected. Other data collection in Temuco and Curacautin proceeded smoothly in local clinics and with community support. 

Panamanian collection (n = 200) occurred during May and June 2004 in Jaguito-El Roble, Tonosi, and Pocri. Unlike the Chilean collection efforts which occurred during short periods of time and relatively quickly, Panama had available resources, infrastructure, and time to invest in implementing the survey. Each site received the appropriate number of surveys with each copy of the survey stamped with the local Ministry of Health approval. The survey was then administered by local nurses and waiting room staff to every third adult who was willing to participate. The researcher then returned to the individual clinics after completion to pick up the surveys. The nurses were asked if there were any adverse events during the implementation. None were reported. 

Unlike Chile and Panama, where survey implementation was met with enthusiasm and willingness to participate, northwestern New Mexico proved to be more problematic and occurred later and over a longer period of time. This could be because of overworked staff, who are required to do more with less, or a general suspicion of yet one more hantavirus researcher in the neighborhood. Eventually, however, with the assistance of a state epidemiologist, three suitable sites in Grants, Farmington, and Gallup were located in 2008 and the surveys were implemented. Once each site completed the requisite number of surveys (n = 200), the information was transmitted to the investigator. No adverse events were reported. 

Finally, for the analyses, there was a reasonable expectation of reliability and power because the minimum number of collected surveys (n = 601) was appropriate for the number of mediating and dependent variables in the model (20 mediating/dependent variables times 10 surveys for each variable times 3 countries). Data entry was performed in EpiData^®^, then imported into SPSS for Windows^®^ for final data analysis. Analyses began with chi-square tests for independence for each variable with cross-country sites, all-risks combined. This method was deemed most appropriate for exploring basic descriptive measurements within and among sites, and in preparation for the next level of analyses. Simple t-tests were then used where appropriate to confirm significance of chi-square tests within country sites, and f-tests were utilized for the same variables across countries to compare the means on continuous variables for the high/low risk groups. To address the hypotheses, logistic stepwise regression models were then constructed upon examination of the descriptive results. While the results were already confirmed if the sample populations was representative of the general population in each site, specific dependent behavioral variables were chosen to address the specific aims of the study. Finally, there will be discussion on the correlations of the age, gender, and occupation with respect to knowledge and significant behavior responses in the combined sites. 

### 2.6. Potential Biases and Study Limitations

Potential biases and study limitations need to be addressed. While this study was exploratory in nature, survey implementation and subsequent data collected was not a true random population samples in each country. A few potential biases also need to be considered in this respondent population. First, Chilean and Panamanian clinics tend to have some sort of hantavirus information already posted on clinic walls. However, it should be noted that data collection in Chile and Panama occurred in 2004, seven years after the first Chilean outbreak, and four years after the Panamanian outbreak, so the likelihood that respondents would have already heard about hantavirus was high. Therefore, the survey was able to adjust by asking when the respondent heard about hantavirus, including learning about hantavirus from medical practitioners as an open-ended question. 

Secondly, the socio-economic status of those in the clinics assumes patients have access to medical care and are already aware of specific diseases and indeed, targeted populations at each site were in health clinics seeking medical attention for some other ailment. Therefore, data collected from clinics has the potential to skew the socio-economic status toward those who have access to medical care. This is especially relevant in northwestern New Mexico, because access to medical care implies higher income (and thus availability of health insurance), higher education and better occupation. 

Third, data were collected over an extended period of time, rather than within a few months of each other. While Chile and Panama officials were eager to assist, clinician and general populations in New Mexico were more reluctant. Perhaps, as mentioned above, for the New Mexicans, the collective memory of the 1993 outbreak lingered, with the misinformation, stereotype, and unfounded ethnic innuendo that permeated the first few weeks of the outbreak. Perhaps clinical staff was overwhelmed with more to do with fewer resources and not inclined to engage in the survey process. However, once initial barriers were overcome, it is believed the self-administered nature of the instrument combined with anonymity helped alleviate any fears clinicians may have had. Fourth, migration in and out of high and low risk areas is also a consideration. While the questionnaire asked when they heard about hantavirus, it did not address the “where.” This is something that needs to be addressed in future data collection efforts. 

Fifth, respondents were asked to identify the biome in which they lived, which is not consistent across survey sites and tends to be very subjective. Traditional spaces could be considered rural, rural agriculture, city or suburb, depending upon the respondents’ personal connection to the space in which they live. For example, respondents in Jaguito/El Roble, Panama, considered themselves as residing in the city, yet their homes are butted up to actively used agricultural properties. In Melipeuco, Chile, which was the most rural community surveyed, there are paved streets and homes organized in a central location with agricultural activities occurring on the outskirts, yet to get to the community, one must travel 9.67 km on unpaved roads. 

## 3. Results and Discussion

The following tables and discussion describes levels of concern across sites about contracting hantavirus (Table 4), knowledge and prevention (Table 5), followed by likelihood of behavior changes by age and occupation across sites (Table 6). 

Compared to each other, Table 4 shows that New Mexico, Panama, and Chile demonstrated overall differing levels of concern over contracting hantavirus. While Panama’s respondents were “very concerned”, Chile’s respondents were “concerned”. Perhaps this is a result of increased awareness programs. New Mexico respondents however, revealed the least amount of concern from “a little bit” to “none at all”. While there is no clear explanation for this, and perhaps could be researched further, on possible reason is that New Mexico experiences a varied amount of zoonotic diseases on a daily basis, such as plague, West Nile Virus, *etc*. Another explanation could be that while hantavirus case fatality rates are comparable to Chile’s as shown in Table 1, in the United States, HPS remains relatively rare phenomenon per 100,000 persons. Despite the various levels of concern each country exhibited, the implication is that the majority of the public also desires proper information on how to protect themselves and their families from exposure. If all respondents had no concern at all, public health programs would be moot and ignored entirely. 

**Table 4:** Levels of concern about contracting Hantavirus.

Level of Concern	NWNM (n = 200)	Panama (n = 200)	Chile (n = 201)	Chi-sq (df)	*p*
Concern about catching HPS, No. (%)	Overall chi-sq (df):			129.065 (8)	0.000
Very concerned	36 (18.0)	**85 (42.5)**	81 (40.2)	32.954 (2)	0.000
Concerned	32 (16.0)	62 (31.0)	**68 (33.8)**	18.679 (2)	0.000
Little bit of concern	**32 (16.0)**	28 (14.0)	19 (9.5)	3.956 (2)	0.138
Not much concern	**53 (26.5)**	1 (9.5)	7 (3.5)	88.847 (2)	0.000
Not concerned at all	**30 (15.0)**	16 (8.0)	22 (10.9)	4.924 (2)	0.085

**Table 5:** Knowledge and prevention all-risk sites.

Knowledge/Behavior	NWNM (n = 200)	Panama (n = 200)	Chile (n = 201)	X^2^ (df)	*p*
Contaminated air	176 (88.0)	173 (86.5)	**189 (94.0)**	6.793 (2)	0.033
Mice	176 (88.0)	187 (93.5)	**193 (96.0)**	9.731 (2)	0.008
Mosquitoes Ticks Fleas	**23 (11.5)**	8 (4.0)	1 (0.5)	25.117 (2)	0.000
	**14 (7.0)**	0 (0.0)	0 (0.0)	28.739 (2)	0.000
	**20 (10.0)**	1 (0.5)	0 (0.0)	37.698 (2)	0.000
Mop Disinfect	54 (27.0)	**79 (39.5)**	53 (26.4)	10.276 (2)	0.006
	87 (43.5)	89 (44.5)	**114 (56.7)**	8.704 (2)	0.013
Sweep Dust masks	57 (27.0)	**100 (50.0)**	38 (18.9)	46.351 (2)	0.000
	**49 (24.5**)	24 (12.0)	22 (10.9)	17.104 (2)	0.000

How the messages are received and translated into behavior responses also varies among the populations as shown in Table 5. The most knowledgeable respondents on how hantavirus is transmitted live in Chile, who correctly identified contaminated air and rodents as transmission routes. Further, New Mexican respondents incorrectly identified mosquitoes, ticks, and fleas. This is probably because (as noted above) in addition to hantavirus, New Mexico and the Four-Corners area of the United States also experience problems with West Nile Virus, Rocky Mountain Fever, and plague, each transmitted by arthropod vectors, rather than rodent reservoirs. It is entirely possible that northwestern New Mexico receives mixed messages due to the nature of the *ad-hoc* (publicity as cases emerge) and passive outreach programs through the websites. Also limited funding and resources in New Mexico public health information programs, includes routes of exposure for all of these diseases, so the messages quite possibly is confusing to the general population. Also noted is that a few Panamanians believe mosquitoes transmit hantavirus. This could be because dengue is endemic in Panama and there may be lingering generational memory of malaria and yellow fever encountered during Panama Canal construction. However, the table also shows that all populations engage in some sort of positive and negative risk-reduction cleaning activity. Chileans disinfect the most, which is a positive prevention risk reduction method, but a few continue to sweep and use dust masks. Panamanians mop, which is another positive behavior change, but not before sweeping in likelihood for mopping preparation. New Mexicans have a tendency to wear dust masks, which are ineffective in preventing the virus from entering the body. 

**Table 6:** Behavior change likelihood by age and occupation across sites.

Behavior	Age Groups					
*How habits have changed*	18–25	26–34	35–44	45–54	55–64	>64
Mop more	r = −0.014	r = 0.083 *	r = −0.044	r = −0.067	r = 0.005	r = 0.097 *
Disinfect more	r = 0.024	r = 0.122 **	r =−0.042	r = −0.060	r = 0.023	r = −0.022
Use rubber gloves	r = −0.018	r = 0.007	r = −0.087 *	r = 0.048 *	r = 0.048	r = 0.011
*How purchases have changed*	18–25	26–34	35–44	45–54	55–64	>64
Buy dust masks	r = −0.085 *	r = 0.017	r = −0.021	r = 0.023	r = 0.101 *	r = −0.017
Buy disinfectants	r = −0.041	r = 0.084 *	r = 0.020	r = −0.037	r = 0.036	r = −0.057
**Behavior**	**Occupation**					
*How habits have changed*	Professional	Skilled labor	Agricultural Farmer	Secretary Clerical	Sales	Ama de Casa
Mop more	r = −0.086 *	r = −0.029	r = 0.085 *	r = 0.029	r = −0.009	r = 0.103 *
Disinfect more	r = 0.030	r = −0.012	r = −0.016	r = −0.004	r = −0.029	r = 0.091 *
Use rubber gloves	r = 0.007	r = 0.091 *	r = 0.016	r = 0.099 *	r = −0.055	r = −0.090 *
Use dust mask	r = 0.171 **	r = 0.002	r = −0.010	r = 0.060	r = 0.034	r = −0.146 **
*How purchases have changed*	Professional	Skilled labor	Agricultural Farmer	Secretary Clerical	Sales	Ama de Casa
Buy poison	r = −0.019 **	r = −0.051	r = 0.123 **	r = −0.051	r = 0.007	r = 0.136 **
Buy dust masks	r = 0.083 *	r = −0.034	r = 0.058	r = 0.094 *	r = −0.015	r = −0.135 **
Buy disinfectants	r = 0.137 **	r = −0.070	r = −0.039	r = 0.129 **	r = −0.026	r = −0.028
Buy ventilator masks	r = 0.094 *	r = 0.018	r = −0.021	r = 0.045	r = −0.007	r = −0.047

* *p* < 0.05; ** *p* < 0.01.

Overall, Table 4 and Table 5 demonstrate that in terms of overall levels of concern, knowledge and prevention, Chilean respondents were concerned, but also learned correct information on how hantavirus is transmitted (by air) and by what (rodents), and they clean the best compared to the other two countries. Panamanian respondents were the most concerned, but demonstrated a poor behavior response by sweeping followed by a proper response of mopping. Finally New Mexico respondents, who were the least concerned, not only demonstrated incorrect information on how hantavirus is transmitted, but also did not perform the same level of cleaning practices in relation to the other sites, while wearing ineffective dust masks during sweeping activities. Therefore, results show that respondents in northwestern New Mexico and Panama continue to sweep more than in respondents in Chile. Chileans have a tendency to engage more in proper cleaning methods of disinfecting than those in northwestern New Mexico and Panama. Finally, while public health messages appear to be more effective in Chile, improper cleaning behavior still occurs in all three populations. 

Basic descriptors shown above demonstrate that sweeping still occurs among all populations, yet certain age groups and occupations across populations do exhibit likelihoods of mostly positive behavior changes. Dust masks, which are ineffective protection while cleaning, are more likely to be purchased by younger and older age groups of 18–25 and 55–64. The age group 26–34 exhibits the highest likelihood of engaging in the most activities to reduce potential exposure to hantavirus: they purchase disinfectants, and they mop and disinfect while using rubber gloves. However, with the exception of dust mask purchases, most age groups are likely to engage in some positive activity to proactively protect and promote good health practices indicating outreach activities do have an effect. Because basic descriptors show Chileans are disinfecting more than Panamanians and New Mexicans, the messages appear to be more effective in Chile for most age groups. 

Specific occupational activities also show a likelihood to purchase proper cleaning materials and to use them to reduce potential exposure to hantaviruses. Professionals and ama de casa (housewives) generally will buy disinfectants, masks, use rubber gloves, and buy poison. Professionals were most likely to purchase ventilator masks, but a closer examination would be warranted, because the 2000 New Mexico pilot project also showed farmers purchase ventilator masks with a reasonable expectation that the masks are used during dusty agricultural activities that are not related to hantavirus prevention measures. It should be noted that the purchase and use of dust masks continue to be problematic, indicating that messages should not only demonstrate proper methods, but what is not appropriate. 

## 4. Conclusions

During the mid-20th century, the reduction of infectious diseases occurred through improved sanitation [21,22]. The same premise can be applied to emerging and re-emerging zoonotic diseases such as HPS that human populations face in the 21st century. That is, improved sanitation is the best, most efficient way to reduce exposure to disease, while modern technology aids in the identification and treatment of disease. Examining newly emerging and re-emerging diseases such as hantavirus requires a new paradigm and assumptions that: (1) disease will emerge; (2) populations are not homogenous; and (3) the disadvantaged are more likely to be exposed to disease because they are in closer proximity to higher-risk environments [10]. In addition, hantaviruses tend to have varied virulence, such as Andes Virus that may likely be transmitted from person-to-person on occasion [23]. Choclo Virus in Panama, on the other hand, has sustained a case-fatality rate of approximately 20%, which is low compared to Sin Nombre Virus and Andes Virus. On-going serologic studies within high-risk communities show an endemic level of the virus [15]. Sin Nombre Virus continues to be a rare, yet on-going event in North America with outbreaks continuing to take communities by surprise, such as the Yosemite National Park outbreak in August 2012. 

While the two-step seasonal messages promoted in Chile appear to be more effective and should continue to consistently emphasize proper prevention measures, improper cleaning behavior still occurs in all three populations. Therefore, Hantavirus Pulmonary Syndrome (HPS) prevention messages should continue to not only emphasize mopping, disinfecting, trapping, and peridomestic cleanup, including airing out of rural feed and work sheds prior to entry, but also to target specific communities and populations that are of higher risk of exposure and also at risk of not implementing proper cleaning methods. Levels of concern over contracting hantavirus were highest in Panama which indicates the public wants proper information on how to protect themselves and their families from exposure, but the poor behavior response indicates the messages are not as effective as they should or could be. Respondents in the high risk, rural poor sites in northwestern New Mexico and Panama continue to sweep and vacuum more than in Chile while respondents in Chile have a tendency to engage more in proper cleaning methods of disinfecting and mopping than those in northwestern New Mexico and Panama. As mentioned above, because the likelihood of purchasing dust masks is apparent across populations, a critical component that should be added to HPS messages is what not to do. Messages should also include information that sweeping and vacuuming actually increases exposure risk by stirring up contaminated dust particles. Messages in Northwestern New Mexico and Panama may have an increased effectiveness as evidenced in Chile, if messages can target rural, poor populations. 

While overall the ecology of hantaviruses is better understood, the need for investigating how human populations respond to hantaviruses and how or why people may engage in proper methods to reduce exposure to disease had yet to be researched [8,24]. However, it is believed that by identifying and combining high-risk human populations with rodent reservoir studies, that more effective outreach programs may be developed and outreach plans modified for special population needs. Indeed, targeted messages are the most effective way to inform and educate the public to help elucidate positive behavior change. 

The goal of this project was to assess the effectiveness of hantavirus prevention measures. It is believed that risk reduction of exposure to hantaviruses may be improved through proper cleaning methods and improved household cleanliness via inexpensive, simple methods. Therefore, this study highlights the knowledge and behavior responses to hantavirus in three populations of northwestern New Mexico, Panama, and Chile. While prevention is generally effective across sites, improvements can be made across sites with clear messages targeted to specific groups. Because disease is not random and tends to affect the rural poor with limited resources, messages in New Mexico should emphasize rodent-proofing homes in addition to mopping instead of sweeping. Because respondents in Panama are more likely to sweep prior to mopping, and their housing tends to be in close proximity to agriculture, messages should stress discontinuing sweeping and to mop instead. Chile has devoted a tremendous amount of resources toward positive prevention measures and to a large extent these messages have been very effective. An improvement would be to provide additional information to discontinue wearing dust masks and to increase mopping as well as to continue disinfecting. 

Disease is complex, not random, and will continue to emerge, and in particular hantavirus. Further investigations, such as the one just described, should re-sample the New Mexico, Panama, and Chile populations for time-series analysis. Understanding the roles of human demography, disease ecology, and human responses to disease, calls for a new approach to emerging disease research. To that end, a larger, inter-disciplinary investigation would be the most beneficial approach. The OneHealth concept recognizes the input of various disciplines in positive health outcomes. Biologists and mammalogists contribute through rodent reservoir sampling and analysis, weather and climate change analysis may help predict where sero-positive reservoirs are expanding and/or contracting, thus enabling public health officials target specific at-risk populations with messages tailored to local nuances. 

This was the first study of its kind to address hantavirus outreach programs in the Americas and therefore constrained to the subject. That said, the implications are not limited to hantavirus and indeed similar methods may be applied to other emerging and re-emerging diseases. This type of study is based upon the collective knowledge of scientists from an interdisciplinary perspective, with an end interest in engaging the public in its own health. An example beyond hantaviruses could be investigating issues such as multi-drug resistant tuberculosis or measles. Tuberculosis is often thought of as a problem “elsewhere” but is on the rise. Measles and polio, once thought to be nearly eradicated through vaccines, has shown a dramatic increase with the decrease of vaccinations and ease of long-distance travel. One hospital administrator in Gallup viewed the application of this type of investigation as a way to assess emergency preparedness which will be the key in informing the public with the efficacy of simple, inexpensive prevention measures for emerging zoonotic diseases. Finally, the application of this study may apply to assessing the effectiveness of influenza outreach, such as the emerging H7N9 in China, or other “surprise” diseases, such as MERS in Saudi Arabia. 
